# Editorial for the “Genetic Studies of Fish” Special Issue

**DOI:** 10.3390/genes16070752

**Published:** 2025-06-27

**Authors:** Yang Liu

**Affiliations:** 1 State Key Laboratory of Mariculture Biobreeding and Sustainable Goods, Yellow Sea Fisheries Research Institute, Chinese Academy of Fishery Sciences, Qingdao 266071, China; yangliu@ysfri.ac.cn; 2 Key Laboratory for Sustainable Development of Marine Fisheries, Yellow Sea Fisheries Research Institute, Ministry of Agriculture and Rural Affairs, Qingdao 266071, China; 3 Hainan Innovation Research Institute, Chinese Academy of Fishery Sciences, Sanya 572025, China

Fish represent the most diverse vertebrate group, with over 32,000 species. Genetic studies in fish facilitate research on genetic diversity, germplasm conservation, utilization, and molecular breeding. Multiple techniques, including hybridization, selective breeding, transgenesis, and gene editing, are employed to prevent genetic deterioration [1]. These approaches often yield offspring with enhanced growth rates, survival capacity, and stress tolerance. Integrating multi-omics analyses enables further identification of candidate genes and genetic markers for economically valuable traits. This Editorial highlights key advances from this Special Issue, fostering deeper insights and innovative methodologies in fish genetics.

The main contributions in this Special Issue focus on fish genetic diversity and germplasm preservation. *Microphysogobio rapidus* is an endemic and critically endangered cyprinid fish species exclusively found in the Nakdong River of Korea. To investigate the impact of dam development on the population structure of *M. rapidus*, 10 microsatellite markers were utilized to assess the genetic diversity of the species in the upper Nam, lower Nam, and Deokcheon Rivers. The observed allelic heterozygosity revealed that all three fish groups significantly deviated from the Hardy–Weinberg equilibrium. Additionally, BOTTLENECK analysis and estimated M-ratio values (0.341–0.372), using LDNe software ver. 1.0 [2], indicated a reduction in past population size for all examined groups. Similarly, the population diversity of *M. longidorsalis*, which is endemic to South Korea and inhabits the Hangang River water system, was analyzed using 19 microsatellite loci across six geographical populations. A relatively low genetic diversity value (0.741–0.779) was observed compared to other freshwater fishes. Based on microsatellite polymorphism values (0.007–0.041), the analyzed populations exhibited low genetic differentiation. Therefore, conservation efforts are necessary to enhance fish population diversity in Korean water systems [3]. Meanwhile, 13 highly polymorphic EST-SSR loci were selected to assess the genetic variation in Pacific abalone (*Haliotis discus hannai*) in six populations collected from China [4]. The cultivated population outside the Changshan Islands showed a 22.79% reduction in allele diversity compared to the wild population. Pairwise Fst value analysis revealed significant population differentiation in most populations, with the greatest difference observed in the cultured populations (Fst = 0.1334).

Redundancy analysis was conducted to identify SNPs associated with environmental adaptations in sockeye salmon populations along the West Pacific Coast. Specifically, candidate loci (*aldob-135*, *hgfa*, and *rag3-93*) involved in immune response and ion homeostasis were found to play a critical role in genetic adaptation to spawning watershed conditions [5]. During the migration of Atlantic salmon from rivers to the ocean, they undergo a key developmental process called smoltification. This period is characterized by mild immunosuppression; however, the B cell repertoire shows an increasing trend in terms of cell proliferation and migration. Enhanced trafficking and renewal of B cells contribute to the survival of virus-infected fish by secreting immunoglobulins [6,7]. These findings underscore a novel role of B cells in the anadromous migration of salmonids.

In aquaculture, the cryopreservation of sperm not only helps maintain high-quality germplasm but also facilitates cross-breeding among fish [8]. Despite this, comprehensive studies on the damage and response mechanisms of long-term frozen fish sperm are scarce. A transcriptome analysis identified four candidate transcripts (*scarb1*, *odf3*, *exoc8*, and *atp5f1d*) related to mitochondria and flagella as response genes for grouper sperm freezing. The heterosis of economically important traits in cultured fish has enhanced aquaculture production for centuries, yet its genetic and molecular foundations remain unclear [9,10]. Recently, a new hybrid sturgeon germplasm (*Acipenser schrenckii* ♂ × *A. baerii* ♀) was used to explore the mechanisms of hypoxia tolerance. A meta-analysis revealed that the HIF pathway was activated following hypoxia exposure, with significant expression of *hif-1α* and *hif-3α* in the gills, showing over 20-fold changes and indicating their crucial roles in the hybrid sturgeon’s adaptation to low-oxygen stress [11].

The use of genome-editing technology has the potential to further boost aquaculture production, but a major challenge lies in identifying target genes to achieve desired phenotypes [12]. This review discusses candidate genes for genome editing that influence body development, growth, pigmentation, and sex determination in five species from the Salmonidae and Cyprinidae families. Notably, the most relevant genes for aquaculture include *mstnba*, *pomc*, and *acvr2*, which enhance muscle growth; *runx2b*, whose knockout prevents bone formation in myoseptae; and *dnd*, *mettl3*, and *wnt4a*, which lead to sterility when nonfunctional [13,14,15]. Somatolactin alpha (SLα), a hormone specific to fish that regulates body color, was studied using CRISPR/Cas9 to identify its receptor (SLR) in medaka (*Oryzias sakaizumii*). SLR mutants were created and showed premature lethality after hatching, highlighting the receptor’s critical role in growth hormone signaling and normal development [16].

Despite the challenges faced in applying molecular breeding technology for large-scale fish production, we believe that ongoing collaboration between academia and industry is essential for overcoming these obstacles.
